# Incidence and Risk Factors for Long-Term Persistence of Diastolic Dysfunction after Aortic Valve Replacement for Aortic Stenosis Compared with Aortic Regurgitation

**DOI:** 10.3390/jcdd10030131

**Published:** 2023-03-20

**Authors:** Luminița Iliuță, Andreea Gabriella Andronesi, Alexandru Scafa-Udriște, Bogdan Rădulescu, Horațiu Moldovan, Florentina Ligia Furtunescu, Eugenia Panaitescu

**Affiliations:** 1 Medical Informatics and Biostatistics Department, University of Medicine and Pharmacy “Carol Davila”, 050474 Bucharest, Romania; 2 Cardioclass Clinic for Cardiovascular Disease, 031125 Bucharest, Romania; 3 Nephrology Department, University of Medicine and Pharmacy “Carol Davila”, 050474 Bucharest, Romania; 4 Nephrology Department, Fundeni Clinical Institute, 022328 Bucharest, Romania; 5 Department of Cardio-Thoracic Pathology, University of Medicine and Pharmacy “Carol Davila”, 050474 Bucharest, Romania; 6 Department of Cardiology, Clinical Emergency Hospital, 014461 Bucharest, Romania; 7 Department of Cardiovascular Surgery, Emergency Institute for cardiovascular diseases “C.C Iliescu”, 022328 Bucharest, Romania; 8 Academy of Romanian Scientists (AOSR), 3 Ilfov Street, 050044 Bucharest, Romania; 9 Department of Cardiovascular Surgery, Clinical Emergency Hospital, 014461 Bucharest, Romania; 10 Department of Public Health and Management, University of Medicine and Pharmacy “Carol Davila”, 050474 Bucharest, Romania

**Keywords:** restrictive diastolic filling pattern, aortic valve replacement, aortic stenosis, aortic regurgitation, systolic dysfunction, postoperative diastolic dysfunction

## Abstract

(1) Background: Severe left ventricular (LV) diastolic dysfunction with a restrictive diastolic pattern (LVDFP) is generally associated with a worse prognosis. Its evolution and reversibility in the short- and medium-term after aortic valve replacement (AVR) has been little-studied. We aimed to evaluate the evolution of LV remodeling and LV systolic and diastolic function after AVR in aortic stenosis (AS) patients compared to aortic regurgitation (AR). Moreover, we tried to identify the main predictive parameters for postoperative evolution (cardiovascular hospitalization or death and quality of life) and the independent predictors for the persistence of restrictive LVDFP after AVR. (2) Methods: A five-year prospective study on 397 patients undergoing AVR for AS (226 pts) or AR (171 pts), evaluated clinically and by echocardiography preoperatively and until 5 years postoperatively. (3) Results: 1. In patients with AS, early post AVR, LV dimensions decreased and diastolic filling and LV ejection fraction (LVEF) improved more rapidly compared to patients with AR. At 1 year postoperatively, persistent restrictive LVDFP was found especially in the AR group compared to the AS group (36.84% vs. 14.16%). 2. Cardiovascular event-free survival at the 5-year follow-up was lower in the AR group (64.91% vs. 87.17% in the AS group). The main independent predictors of short- and medium-term prognosis after AVR were: restrictive LVDFP, severe LV systolic dysfunction, severe pulmonary hypertension (PHT), advanced age, severe AR, and comorbidities. 3. The persistence of restrictive LVDFP after AVR was independently predicted by: preoperative AR, the E/Ea ratio > 12, the LA dimension index > 30 mm/m^2^, an LV endsystolic diameter (LVESD) > 55 mm, severe PHT, and associated second-degree MR (*p* < 0.05). (4) Conclusions: AS patients had an immediate postoperative evolution in terms of LV remodeling, and LV systolic and diastolic function were more favorable compared to those with AR. The restrictive LVDFP was reversible, especially after the AVR for AS. The main prognostic predictors were the presence of restrictive LVDFP, advanced age, preoperative AR, severe LV systolic dysfunction, and severe PHT.

## 1. Introduction

The presence of a severely impaired diastolic function with a restrictive left ventricular (LV) diastolic filling pattern (LVDFP) determines a more unfavorable prognosis in any cardiac disease [1,2,3]. Although there are many studies on diastolic dysfunction, there is little information on diastolic dysfunction in the postoperative cardiac surgical patient undergoing valve replacement [4,5,6]. There are only a few studies that have evaluated the influence of the restrictive LVDFP on the postoperative course, but these are mostly heterogeneous, with relatively small populations and variable patient selection, being difficult to compare between them to draw a unified conclusion [1,2,3,7,8,9].

Diastolic dysfunction has been demonstrated to precede the alteration in systolic function in patients with LV hypertrophy due to aortic valve disease. 

After successful aortic valve replacement (AVR) in aortic stenosis (AS), the early postoperative evolution is marked by an immediate hemodynamic improvement by reducing the pressure overload, with a significant regression of LV hypertrophy and the LV mass index/LV end-diastolic volume ratio [10]. This evolution is mainly due to the regression of the muscle tissue, while the total amount of the nonmuscular tissue (fibrous tissue) of the LV remains unchanged and is associated with the deterioration of LV diastolic function. Thus, depending on the amount of fibrous tissue, recovery of LV systolic and diastolic function in patients with AS is progressive and may continue for decades after AVR. That is why the degree of extension of irreversible interstitial fibrosis and the LV architecture are very important in postoperative remodeling, but it is very difficult quantify before surgery [11]. Therefore, it is very important to find clinical and echocardiographic parameters that can be taken into account for preoperative risk assessment and in order to avoid a too-late indication for AVR, with a negative effect on the prognosis.

On the other hand, considering patients with severe chronic aortic insufficiency (AR) undergoing AVR, the postoperative evolution is different. In the first few months after surgery there is a marked decrease in the LV dimensions, accompanied by a significant increase in the LV systolic performance [3,4,5]. Nevertheless, in some patients with preoperative LV dysfunction, despite the correction of valvular insufficiency, valve replacement leads to a smaller reduction in LV diastolic volume and a minor improvement in LV systolic function. Preoperative identification of these patients using imaging tools remains a challenge. 

The patients with severely altered diastolic performance before AVR (either for AS or AR) have a more tedious recovery. The preoperative identification of patients with evidence for irreversible myocardial dysfunction with conventional echocardiographic examination remains difficult. Routine preoperative measurements of ejection fraction (EF) and LV dimensions as an index of the LV function cannot accurately predict how postoperative LV performance will evolve. Thus, they are not sufficient enough in making clinical decisions and for recommending surgery in patients with aortic valve disease [12,13,14,15]. 

On the other hand, serial long-term studies on the effect of AVR on diastolic function and the relation between the reversal of LV dilatation and the increase in LV systolic and diastolic performance has not been reported. Moreover, there are no studies which compared patients with AS to patients with AR undergoing AVR regarding the influence and evolution of the diastolic filling pattern. To address these issues, we studied a series of patients undergoing AVR for chronic AR and AS with preoperative and serial long-term postoperative echocardiographic and clinical evaluations.

### Objectives

The first objective of this study was to evaluate the evolution of left ventricular (LV) remodeling and LV systolic and diastolic function after AVR, comparing patients with preoperative AS to patients with preoperative AR. In addition, we tried to define the parameters which can be taken into consideration as independent predictors for immediate- and medium-term evolution (in terms of cardiovascular hospitalization or death and quality of life) in AS compared to AR patients, and their adjusted value for identifying a high-risk group. Finally, we tried to establish the independent predictors for the persistence of the restrictive LVDFP after isolated AVR in patients with AS or AR.

## 2. Materials and Methods

### 2.1. Study Population

A total of 410 patients met the criteria for inclusion (surgical aortic valve disease) in the study, but 13 were excluded due to exclusion criteria (9 patients with associated significant coronary lesion, 1 patient with postoperative prosthesis mismatch, and 3 patients who required a postoperative pacemaker implantation). The study was prospective and finally included 397 patients undergoing AVR for AS or AR who were followed-up clinically and by echocardiography with a median duration of 6.1 years (between 1st of January 2015 and 1st of January 2022). Only 5 patients were lost at the mid-term follow-up visits.

All patients signed the written informed consent and were required to attend the follow-ups. The protocol of the study was approved by the designated ethics committee.

Patients were assessed by clinical examination and by echocardiography before surgery and after surgery at 10 days, 1, 3, and 6 months, and yearly thereafter until 5 years. 

#### Exclusion Criteria

- Previous or associated mitral or tricuspid valve replacement or repair;
- Aortic dissection;
- Acute endocarditis;
- Congenital disease unrelated to AR or AS;
- Coronary significant lesions (more than 50% stenosis);
- Chronic atrial fibrillation;
- Left or right bundle-branch block;
- Postoperative prosthesis mismatch;
- Pacemaker implantation.

We did not exclude patients undergoing concomitant procedures associated with AVR, such as ascending aortic surgery.

According to the hospital’s preoperative evaluation protocol for AVR, coronarography was performed in all patients over 35 years of age, as well as in patients under 35 years with angina pectoris. We excluded patients with associated coronary artery disease (>50% reduction in luminal diameter of any coronary artery).

### 2.2. Ultrasound Methods

For echocardiographic examinations, we used a Philips Affinity 30 or a portable General Electric VIVID machine with a 3.5 MHz probe. For measurements, we followed the recommendations of the European and American Society of Echocardiography [16,17].

The main echographic parameters assessed before surgery were: LV end-diastolic and end-systolic volume and diameter, left atrium (LA) diameter and indexed volume, LV systolic and diastolic function (including tissue doppler imaging—TDI—evaluation), and hemodynamic parameters for AS and AR severity evaluation. Dimensions of the heart diameters and wall thickness were measured by 2-dimensional echocardiography with M-mode guiding. 

For the diagnosis of surgical AS, we used the hemodynamic parameters primarily recommended for evaluation: AS jet velocity, mean transaortic gradient, and the valve area by continuity equation. The degree of AR (grade 3 or 4) was determined by composite analysis of either continuous Doppler or color flow Doppler.

At postoperative visits, we measured also the dimensions of the heart chambers, LV systolic and diastolic performance, and the hemodynamic parameters of the aortic prosthesis [18]. Echocardiographic measurements and clinical evaluations were collected and electronically transferred in a dedicated application.

LV systolic performance was appreciated by the calculation of the LVEF using the volumetric Simpson’s method. LV diastolic filling was evaluated using a comprehensive echocardiographic study for the assessment of diastolic function integrating all available parameters from two-dimensional, Doppler, and Tissue Doppler examination [18,19]. We used the traditionally echocardiographic evaluation of diastolic function by measuring the transmitral-flow parameters and assessing the early (E) and late (A) diastolic filling velocities, the E/A ratio, and the E-wave deceleration time (EDT).

For TDI, using the sample volume placed at the lateral border of the mitral annulus in the apical four-chamber view, we measured: the peak annular systolic velocity (Sa), early diastolic velocity (Ea), and late-diastolic (Aa) velocity.

The restrictive LVDFP was considered to be present if either of the following echographic findings were found: E/A ratio > 2 or EDT < 150 msec or IVRT < 60 msec and Ea/Aa < 1 with elevated filling pressures (E/Ea ratio > 12) [20].

### 2.3. Follow-Up Visits

All patients were assessed postoperatively by clinical examination, echocardiography, and blood tests. Laboratory parameters evaluated at each visit included: blood tests (platelet count, hemoglobin, hematocrit, aminotransferases, lactic dehydrogenase). When we had the suspicion of heart failure with preserved systolic function, brain natriuretic peptide (BNP) dosing was done. Diastolic heart failure was considered unlikely if the BNP was less than 35 pg/mL and possible for values higher than 100 pg/mL.

In order to evaluate the global quality of life, a self-reported questionnaire was used for both the mental component (MCS) and the physical one (PCS). The scores ranged from 0 (lower) to 10 (higher quality of life). Patients evaluated their change in quality of life at the 30-day follow-up by answering the question: “How would you rate your quality of life now?”. The answers they had to choose from were: “Worse than before your surgical procedure”, “The same as before your surgical procedure”, and “Better than before your surgical procedure”.

Depending on the type of the aortic lesion, patients were divided in two groups:

- Group A—226 pts undergoing AVR for AS;
- Group B—171 pts undergoing AVR for AR.

AVR was performed with a bileaflet mechanical valve in 70.35% of patients from the AS group and in 71.93% of patients from the AR group. For the rest of the patients, valve replacement was done with a bioprosthetic valve.

### 2.4. Statistical Analysis

Data were analyzed using Statistical Package for the Social Sciences 23 (SPSS 23) software.

For comparison of preoperative and postoperative echocardiographic categorical variables, we used the Pearson chi-square test, likelihood ratios, and Fisher’s exact test. Qualitative data were recorded as percentages. Numerical continuous variables were expressed as mean ± standard deviations. Normal distribution of all continuous variables was tested with an Independent Samples T-Test and for three groups with ANOVA. LVEF was used as a continuous and discrete variable (low-LVEF versus normal-LVEF). Baseline continuous variables were compared among the two LVEF groups (low-LVEF versus normal-LVEF) by means of paired t-tests and ANOVA. Moreover, a one-way ANOVA for repeated measures (with a Greenhouse–Geisser correction) was used for comparing preoperative, early postoperative, and late postoperative data from patients with AR and patients with AS. If the analysis showed a significant difference, the Scheffé procedure was applied. 

We used univariate logistic regression analysis in order to compare the two groups. Moreover, the association between preoperative data and the magnitude of postoperative change in the LV dimensions and function was tested by linear regression analysis. 

The parameters that were found statistically significant in the univariate regression analysis were entered in the multivariate regression models. In order to identify variables associated to predictive factors of mortality, we used multivariate logistic models, taking into account baseline echocardiographic characteristics. The Cox and Snell/Nagelkerke value was calculated in order to evaluate goodness of fit of the model. A *p* < 0.05 was considered statistically significant.

Overall survival and freedom-from-events (death, NYHA class, and quality of life) after the AVR were estimated by use of the Kaplan–Meier method. Multivariate Cox proportional hazards models were fit to identify variables associated with long-term outcome. Relative risks/odds ratios, hazard ratios, and 95% CIs were reported. 

The main null hypotheses tested were:

1. Is the restrictive flow pattern reversible mostly after AVR for AS than AR, both in the early postoperative period and on medium-term?
2. Are early and medium-term prognosis and LV remodeling after AVR in preoperative restrictive LVDFP patients better in those with AS compared to those with AR?
3. Are severe AR, restrictive LVDFP, and severe pulmonary hypertension (PHT) independent predictors for unfavorable postoperative evolution in patients undergoing AVR?

The testing of the statistical hypotheses was done with the help of univariate and multivariate logistic regression analysis and the calculation of the correlation coefficient. Moreover, to evaluate the differences between the three moments analyzed (preoperative, early postoperative, and late-postoperative), we used the nonparametric Friedman test.

## 3. Results

The demographic characteristics of the two studied groups showed a higher average age and a higher percentage of men in the AS group. The percent of the patients with comorbidities and the mean LVEF was similar in the two studied groups. In the AR group, there was a significantly higher percentage of patients with NYHA class IV and with a restrictive LVDFP (Table 1).

**Figure 1:**
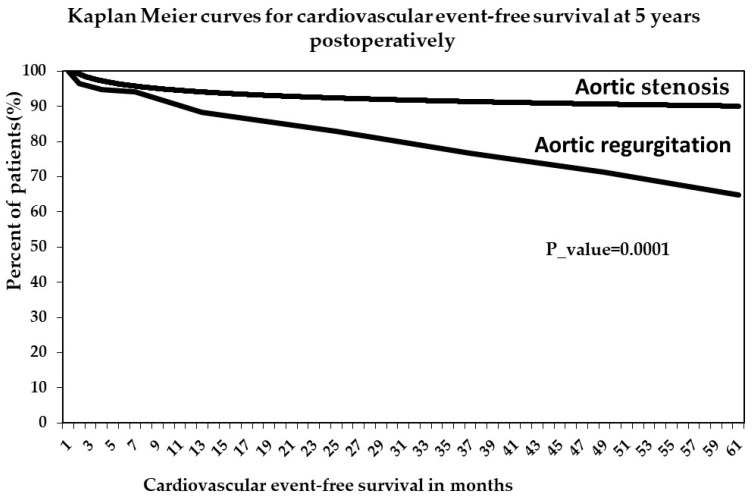
Kaplan–Meier curves for cardiovascular event-free survival at 5 years postoperatively.

Patients with preoperative AR had a more severe evolution after surgery, with a higher mortality rate and more cardiovascular events than those with AS (Figure 1). At 5 years postoperatively, cardiovascular event-free survival, including hospital visits caused by heart failure symptoms and sudden cardiac death, was significantly higher, with almost 22 percent in the group of patients with preoperative AS (87.17%) compared with the AR group (64.91%), with a significant *p*-value (0.0001).

The main independent predictors for death and hospitalization for HF at 5 years postoperatively in patients with aortic valve disease undergoing AVR revealed by univariate logistic regression analysis and the associated RR (95% CI) were: restrictive LVDFP (RR = 6.8 (5.9, 7.6), *p* = 0.001), preoperative AR (RR = 3.12 (2.57, 3.74), *p* = 0.0001), severe preoperative LV systolic dysfunction with an LVEF less than 35% (RR = 2.7, *p* =0.035), severe preoperative PHT with mean PAP > 50 mmHg (RR = 2. 36 (2.37, 3.29), *p* = 0.021), patients’ age of more than 75 years (RR = 1.86 (1.74, 2.07), *p* = 0.032), comorbidities (RR = 1.95 (1.67, 2.79), *p* = 0.071), moderate LV systolic dysfunction with LVEF between 35 and 50% (RR = 2.03 (1.51, 2.73), *p* = 0.053), and dilated LV with preoperative LVES diameter > 55 mm (RR = 2.27 (1.65, 2.95), *p* = 0.059).

Moreover, univariate regression analysis showed that the preoperative AR in patients with restrictive LVDFP turned out to be an independent predictor for increasing the mortality rate (*p* = 0.0001), with preoperative AR being more harmful for postoperative evolution in AVR patients.

**Figure 2:**
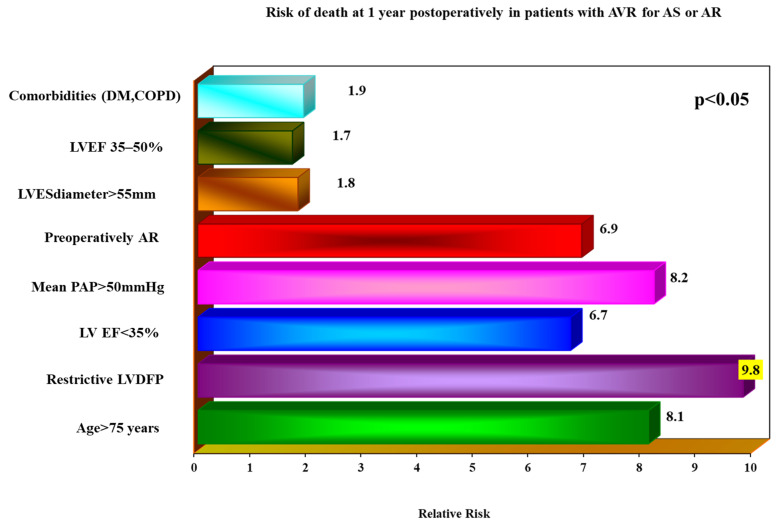
Risk of death at 1 year postoperatively in patients with AVR for AS or AR.

At 1 year postoperatively, the main independent prognostic predictors for the patients with aortic valve disease undergoing AVR revealed by multivariate logistic regression analysis were (Figure 2): restrictive LVDFP (RR = 9.8, *p* = 0.001), severe LV systolic dysfunction with a LVEF less than 35% (RR = 8.7, *p* = 0.002), severe PHT (PHT) with mean PAP > 50 mmHg (RR = 8.2, *p* = 0.021), patients’ age of more than 75 years (RR = 8.1, *p* = 0.013), the presence of preoperative AR (RR = 6.9, *p* = 0.031), comorbidities (RR = 1.9, *p* = 0.071), moderate LV systolic dysfunction with LVEF between 35 and 50% (RR = 1.7, *p* = 0.074), and dilated LV with LVES diameter > 55 mm (RR = 1.8, *p* = 0.067). For regression analysis, in order to identify the prognostic predictors, we used baseline echocardiographic characteristics.

The most important preoperative predictor of early postoperative hospital course and postoperative morbidity was the presence of a restrictive LVDFP. 

In the long term, the logistic regression analysis of the parameters known for increasing the mortality rates after AVR showed that the risk for cardiovascular events was higher in patients with preoperative AR. The main predictors for cardiovascular events at 5 years postoperatively in both the AS and AR groups were LV systolic performance, age, and the presence of preoperative restrictive LVDFP. 

The predictive value for cardiovascular events at 5 years postoperatively of the LV systolic dysfunction, advanced age, or of the presence of a restrictive LVDFP was higher in the AR group of patients. In these patients, the risk for cardiovascular events at 5 years was significantly increased in patients with LVEF < 35% (RR = 8.8, *p* < 0.05), in patients older than 75 years (RR = 7.1, *p* < 0.05), and in patients with restrictive LVDFP (RR = 8.1, *p* < 0.05). 

**Figure 3:**
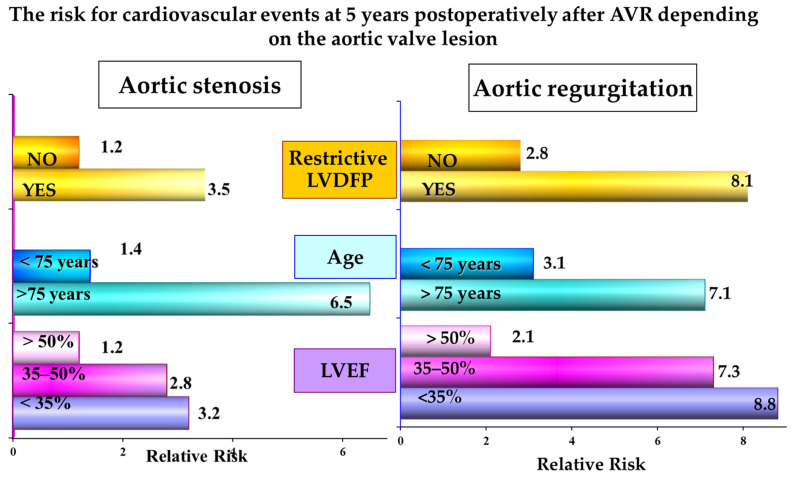
Risk for cardiovascular events at 5 years postoperatively after AVR depending on the type of aortic valve lesion.

In patients with preoperative AS, the relative risk values for cardiovascular events at 5 years postoperatively associated to LV systolic dysfunction, elderly age, and the presence of restrictive LVDFP were smaller and more homogenous than in those with AR. The main independent predictors for increasing the risk for cardiovascular events were age > 75 years (RR = 6.5, *p* < 0.05), restrictive LVDFP (RR = 3.5, *p* = 0.062), and LVEF < 35% (RR = 3.2, *p* = 0.078) (Figure 3). 

From clinical point of view, in the medium and long term, the percentage of patients with a more favorable evolution regarding the NYHA class and quality of life was higher in patients with preoperative AS, regardless of the LV systolic performance before surgery.

The overall percentage of patients whose self-reported quality-of-life scores (SR QOL score) changed between worse/the same/better (including stratification by self-reported mental, physical, and global) was calculated. The percentage of the patients with a preoperative self-reported quality-of-life score < 5 was similar in the two study groups (55.75% in the AS group vs. 56.72% in the AR group). However, the SR QoL evaluated at the 1-month check-up in the AS group showed an improvement in 77.88 % of the patients as opposed to the AR group, where the SR QoL improved in only 41.52% of the patients. 

**Figure 4:**
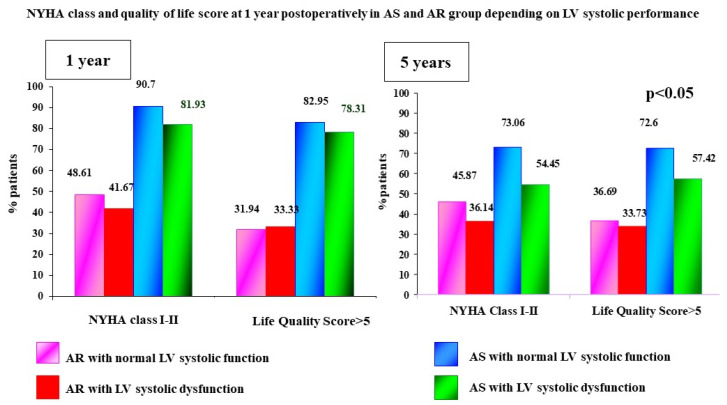
NYHA class and SR QOL > 5 at 1 year postoperatively in AS and AR group depending on LV systolic performance.

Moreover, at the 1-year follow-up, the percentage of patients with NYHA class 1 and 2 was almost two-fold higher in patients with AS in comparison with AR, regardless of the preoperative LVEF (*p* < 0.05) (Figure 4). At the same time, the quality-of-life score > 5 was found in almost 2.3-fold patients with AS compared to patients with AR, both at 1 and 5 years after AVR.

Postoperative echocardiography showed a trend toward improvement for LVEF in AR patients (from 48 ± 4% before surgery to 53 ± 5% 6 months after surgery to 54 ± 6% at 2 years postoperatively, *p* = 0.08) and significant improvement in AS patients (from 50 ± 5% before surgery to 52 ± 12% 6 months after surgery to 55 ± 46% at 2 years postoperatively, *p* = 0.002). 

Postoperative end-diastolic and end-systolic dimensions decreased significantly in both groups, but was early postoperatively mostly in the AS group (Table 2).

Early postoperatively, the evolution of the LV diastolic filling was different in patients with AS rather than those with AR. After the AVR, diastolic filling improved in patients with AS, whereas patients with AR showed a smaller improvement in diastolic filling. Patients with preoperative AR had a more severe postoperative evolution, with a higher rate of persistence of the restrictive LVDFP. At 1 year postoperatively, the percentage of the patients with persistent restrictive LVDFP was 14.16% in the AS group and 36.84% in the AR group (*p* = 0.001). 

The persistence of the restrictive LVDFP has increased the risk of death at 1 year postoperatively (RR = 9.2, *p* < 0.05), regardless of the presence of other known parameters that increased the mortality rate in patients with aortic valve disease undergoing surgical repair. 

Thus, regression analysis revealed that the persistent restrictive LVDFP proved to be an independent predictor for increasing the mortality rate in these patients (*p* = 0.001), regardless of the patients’ age, comorbidities, LV dimensions, and ejection fraction, or the presence of PHT. 

**Figure 5:**
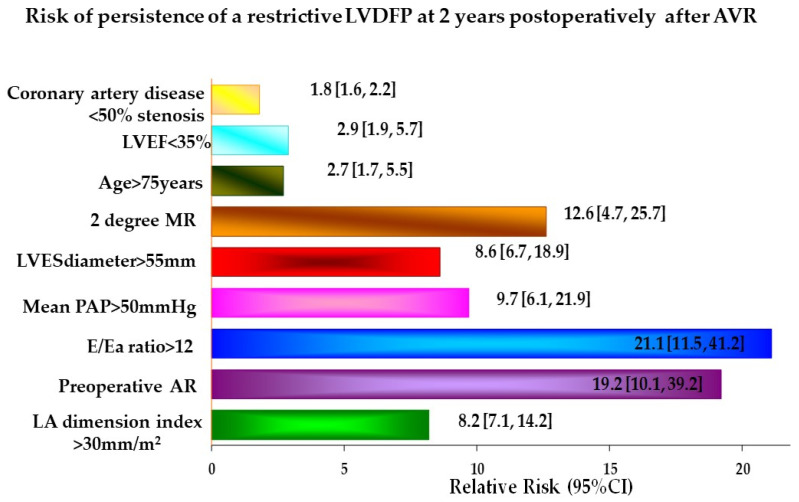
Risk of persistence of a restrictive LVDFP at 2 years postoperatively after AVR.

After the successful AVR, the main independent predictors for the persistence of a restrictive LVDFP revealed by simple linear and multivariate regression analysis were (Figure 5):

- Preoperative AR (RR = 19.2, *p* = 0.0001);
- Severe restrictive LVDFP with an E/Ea ratio > 12 (RR = 21.1, *p* = 0.0001);
- Dilated LA with a an LA dimension index > 30 mm/m2 (RR = 8.2, *p* = 0.0017);
- Severely dilated LV with an LV endsystolic diameter (LVESD) > 55 mm (RR = 8.6, *p* = 0.01);
- Severe PHT with a mean PAP > 50 mmHg (RR = 9.7, *p* = 0.002);
- The presence of an associated second-degree mitral regurgitation (MR) (RR = 12.6, *p* = 0.05).

We found no correlation for the persistence of a restrictive LVDFP after the AVR in these patients for parameters of LV systolic performance, age, or coronary artery disease (Pearson r = 0.45, *p* = NS)

## 4. Discussion

After successful AVR, LV remodeling is different in patients with AS compared with AR due to the relationship between how each type of the valve lesion influences LV geometry and the LV systolic and diastolic functions [20,21]. Our study tried to evaluate how the dimensions of the heart chambers and the systolic and diastolic performance evolved in patients with AS compared to those with AR. The main conclusion was that LV remodeling decreases in postoperative end-diastolic and end-systolic dimensions and improvements in LV systolic and diastolic performance after AVR were earlier in patients with AS compared to AR.

As in our study, other researchers have shown that the early postoperative evolution of patients with AR is usually more unfavorable [7,8]. Moreover, at 1 year postoperatively, the NYHA class and self-reported quality of life in our study groups was found better after the AVR for AS compared to AR, as was reported in other previous studies [22].

Regarding systolic performance, the LVEF showed a tendency to improve both in patients with AS and in those with AR after the AVR [22,23]. In most patients with severe chronic AR, there is an important reversal of LV dilatation, associated with a significant increase in LV systolic performance during the first few months after the AVR. These changes correlate with survival in the next 4–5 years. Thus, patients with normal early postoperative systolic and diastolic performance have an excellent prognosis, while survival rates are reduced in patients with persistent LV dysfunction [22].

Regarding the evolution of the diastolic function postoperatively, the situation is different and depends on the type of valvular lesion (AS or AR) and on the time from surgery. Most studies have shown that, in the majority of patients with AS, LV diastolic function returned to normal after the valve replacement [24,25,26]. In contrast, in AR, diastolic dysfunction with abnormal filling is maintained in a significant percentage of patients after surgical correction, being normalized late after surgery [27,28,29].

However, the presence of a restrictive diastolic pattern determined a more unfavorable postoperative evolution both in patients with AS and in patients with AR. Severe diastolic dysfunction was identified by regression analysis as an independent risk factor for death and hospitalization for heart failure, as other studies have shown [1,5,10,28] [30,31]. Therefore, we also sought to assess whether preoperative restrictive diastolic filling precluded postoperative LV remodeling and improved function, and thus might prove useful in identifying patients who benefit less from AVR. One of the most common problems is that pure diastolic dysfunction is rare before surgery, often occurring together with some degree of systolic dysfunction.

We reviewed the current literature in search for criteria that could be applied to help the preoperative assessment and to improve the postoperative management. In patients with preserved LVEF, restrictive filling usually indicates severe myocardial disease. In clinical practice, restrictive filling is strongly predictive of mortality, especially if it is not reversible after treatment [27,32]. Diastolic dysfunction proved to be a more important predictor of mortality and unfavorable postoperative evolution compared to LV dilation and severe systolic dysfunction both in patients with AS and in those with AR [30,31,32].

The question remains: are the early and medium-term prognoses and LV remodeling after AVR in preoperative restrictive LVDFP patients better in those with AS compared to those with AR? Our study concludes that the improvement of the diastolic performance after surgery was better in AS patients compared to AR patients. In patients with severe AR, diastolic dysfunction persists late after successful AVR, despite recovery of LV systolic performance.

On the other hand, the relevance of LV systolic and diastolic performance as predictors of prognosis and postoperative evolution is different in AR [33]. The importance of diastolic dysfunction for the postoperative evolution in patients with AR is still underestimated; therefore, it is not included in the preoperative risk scores. Moreover, in patients with HF, restrictive diastolic dysfunction is a stronger predictor of mortality than LVEF, and the severity of diastolic dysfunction correlates better than systolic performance with exercise capacity limitation, morbidity, and mortality. Kim et al. also showed that the E/e′ ratio was significantly associated with adverse outcomes in patients with chronic severe AR undergoing AVR and may be useful as a prognostic marker in such patients [34]. These data are consistent with our findings and support the importance of a preoperative assessment of LV diastolic function by TDI [35]. Future, randomized studies on larger groups of patients may clarify this issue and determine if it is appropriate to introduce the restrictive diastolic pattern as a severity parameter in the calculation of the preoperative risk score.

Moreover, knowledge is still limited regarding immediate and long-term postoperative outcome in patients with restrictive LVDFP [26,27,28]. That is why another important question we tried to answer in our study was: does the reversibility of the restrictive flow pattern after AVR depend on the type of the aortic lesion? We concluded that restrictive LVDFP is reversible especially after AVR for AS and not for AR.

In order to answer this question, we assessed the independent predictors for the persistence of the restrictive LV diastolic filling pattern after an isolated AVR. There are few studies evaluating the influence and reversibility of the impaired diastolic function in the early and medium-term postoperatively when comparing AS with AR [8,9]. In our study, we found that LVEF had only a limited predictive value in multivariate models, which included ultrasound parameters for the assessment of diastolic function and PHT. This may highlight the finding that the prognostic information obtained from the assessment of systolic function is insufficient and many other covariates included in the multivariate model are needed. Moreover, we found that the main predictors for the persistence of a restrictive LVDFP in the medium term were E/Ea ratio > 12, preoperative AR, LA dimension index > 30 mm/m^2^, severely dilated LV, severe PHT, and the presence of an associated second-degree MR.

We also tried to establish the main independent predictors for unfavorable postoperative evolution after AVR. The most important predictors were severe AR, restrictive LVDFP, and severe PHT. In the long term, the evolution in AS was significantly influenced only by age, while in those with AR were influenced by age, restrictive pattern, and severe LV systolic dysfunction.

As with other studies, our research showed that early and late results after AVR are affected by the presence of PHT, which is also an important risk factor when we talk about the preoperative evaluation [35,36]. In patients with severe PHT, postoperative mortality after AVR was significantly increased, representing an independent risk factor for reduced quality of life and long-term survival. However, although the postoperative mortality of patients with aortic valve injury and severe PHT is higher, other studies demonstrate that the prognosis of conservatively treated patients is very poor, and AVR improves this prognosis [36]. Thus, the indication for surgical intervention is maintained even in the presence of PHT, but its value should be taken into account in the preoperative evaluation. In our study, a pulmonary artery pressure greater than 50 mmHg was an independent predictor for early postoperative mortality.

Our study only evaluated the short- and medium-term evolution of patients with surgical AVR without taking into account the increasingly important population of those who benefit from transcatheter valve implantation (TAVI). Taking into account that multiple hospitalizations after TAVI are common and are often caused by HF, there are few studies trying to evaluate predictors and prognostic implications of new hospitalizations in the long term after TAVI [37,38]. However, substantial work is needed to obtain a standardized score that includes a limited number of variables that can be easily assessed during routine preoperative assessment and to test the predictive value of the models.

Our study attempts to formulate an update of the concept of preoperative evaluation in surgical aortic valve lesion, but an update of the current knowledge regarding the impact of these factors on functional capacity, morbidity and mortality is still needed. Severe diastolic dysfunction proved to be a better and more sensitive predictor of unfavorable postoperative evolution and mortality than LVEF in both AS and AR.

Since the treatment of postoperative diastolic HF remains difficult and often unsatisfactory, and the postoperative evolution of patients with a restrictive diastolic pattern is often unfavorable, the preoperative recognition of severe diastolic dysfunction is an important prognostic sign and should be incorporated into the calculation of the preoperative risk score. Thus, for the better management of patients with surgical AS or AR, future studies are needed to establish an updated preoperative algorithm that takes into account multiple risk factors, including complete echocardiographic evaluation, which would be useful to be incorporated into current guidelines. 

### Study Limitations

Although this is one of the largest studies to date dealing with the TDI evaluation of diastolic filling in surgical aortic valve disease, the number of the patients was still average.

Moreover, the AVR was done by surgery in all of the patients included in the present study. Therefore, we did not have a comparison group with transcatheter aortic valve implantation.

## 5. Conclusions

1. Postoperative LV remodeling, improvement in systolic and diastolic performance, NYHA class, and quality of life after AVR was faster in patients with AS compared with those with AR.
2. The independent predictors for increased mortality and hospitalization for HF in the medium term in patients with AVR were: restrictive diastolic filling, preoperative AR, advanced age, LVEF < 35%, severe PHT, and the presence of comorbidities.
3. The persistence of the restrictive diastolic pattern in the medium term after the AVR was more frequent in patients with AR, severe LV or LA dilatation, severe PHT, and associated second-degree MR.

Preoperative stratification of the patients undergoing AVR needs to take into account the diastolic function, presence of the PHT, the dimensions of the LV, and the systolic performance of the LV. 

## Figures and Tables

**Table 1:** Baseline patients’ characteristics—AS and AR patients.

	Group A—226 Pts AS	Group B—171 Pts AR	*p*-Value (Test)
Mean (SD) age (years)	72 (12)	68 (11)	0.386014
Women	81 (35.84%)	68 (39.77%)	0.581801
Mean (SD) heart rate	68 (17)	81 (17)	0.645900
Diabetes mellitus, no (%)	11 (4.87%)	9 (5.26%)	0.355588
COPD, no (%)	12 (5.31%)	10 (5.85%)	0.591229
Mean (SD) LVEF (%)	52 (13)	51 (14)	0.0376032
LVEF < 50%, no (%)	89 (39.38%)	69 (40.35%)	0.432215
NYHA class I/II, no (%)	148 (65.49%)	105 (61.40%)	0.001457
NYHA class III, no (%)	64 (28.32%)	40 (23.39%)	
NYHA class IV, no (%)	14 (6.19%)	26 (36.61%)	
Restrictive LVDFP	78 (34.51%),	77 (45.03%)	0.0123004

SD—standard deviation; COPD—chronic obstructive pulmonary disease (≥Gold 2); 1 LVEF—left ventricle ejection fraction; LV—left ventricle; LVDFP—LV diastolic filling pattern; NYHA—New York Heart Association.

**Table 2:** Comparison of preoperative and postoperative echocardiographic variables.

Echographic Variables	Group A—226 Pts with AS				Group B—171 Pts with AR			
	Before Surgery	6 Months after Surgery	2 Years after Surgery	*p*-Value (Test)	Before Surgery	6 Months after Surgery	2 Years after Surgery	*p*-Value (Test)
LV end-diastolic diameter (mm)	54 ± 6	51 ± 9	47 ± 8	0.082321	65 ± 6	62 ± 8	59 ± 7	0.126342
LV lateral wall thickness (mm)	17 ± 0.2	15 ± 0.2	12 ± 0.2	0.043672	14 ± 0.2	13 ± 0.4	12 ± 0.2	<0.001
IVS thickness (mm)	17.0 ± 0.4	15.4 ± 0.5	12.7 ± 0.7	0.052345	13.0 ± 0.4	12.0 ± 0.2	11.4 ± 0.4	<0.001
Mean (SD) LVEF (%)	50 (5)	52 (12)	55 (10)	0.054323	48 (4)	53 (5)	54 (6)	0.082445
EDT (msec)	179.95 ± 60	184.72 ± 65	230.35 ± 74	0.051332	162 ± 6	171 ± 8	177 ± 7	0.063245
IVRT (msec)	119.5 ± 74	120 ± 44	123 ± 48	0.042812	97 ± 2	91 ± 4	83 ± 2	0.031843
E/A	1.6 ± 0.4	1.54 ± 0.5	1.47 ± 0.7	0.071254	1.9 ± 0.3	1.8 ± 0.2	1.9 ± 0.4	0.042345
Ea/Aa	1.2 ± 0.2	1.31 ± 0.3	1.38 ± 0.2	0.084728	1.1 ± 0.3	1.2 ± 0.3	1.3 ± 0.4	0.072672
E/Ea	8.9 ± 1.2	8.41 ± 0.9	7.92 ± 0.4	0.064319	9.7 ± 1.3	9.5 ± 1.3	8.9 ± 1.4	0.062531

LV—left ventricle; IVS—interventricular septum; LVEF—left ventricle ejection fraction; EDT—E-wave deceleration time; IVRT—isovolumetric relaxing time.

## Data Availability

All data generated or analyzed during this study are included in this published article.

## Abbreviations

AS	aortic stenosis
AVR	aortic valve replacement
LV	left ventricle
LVDFP	left ventricle diastolic filling pattern
LA	left atrium
LVEF	left ventricle ejection fraction
LVESD	left ventricle end-systolic diameter
NYHA	New York Heart Association
TDI	tissue doppler imaging
LVEDV	left ventricle end-diastolic volume
LVEDD	left ventricle end-diastolic diameter
EDt	deceleration time
IVRT	isovolumetric relaxation time
LVOT	left ventricle outflow tract
Sa	peak annular systolic velocity
Ea	early diastolic velocity
Aa	late diastolic velocity
BNP	brain natriuretic peptide
ANOVA	analysis of variance
COPD	chronic obstructive pulmonary disease
SR QOL	self-reported quality of life
IVS	interventricular septum
ROC	receiver operating curve
PAP	pulmonary arterial pressure
